# Glycan masking in immunogen design: computational and experimental methods

**DOI:** 10.3389/fimmu.2025.1726810

**Published:** 2026-01-07

**Authors:** Gustavo Araiza, Josuel Morel, Minh H. Tran, Shan Jiang, Sean Murray, Emika Miyamoto, Hannah Vogts, Kyle L. Brown, Jens Meiler, Cristina E. Martina

**Affiliations:** 1 Center for Structural Biology, Vanderbilt University, Nashville, TN, United States; 2 Chemical and Physical Biology Program, Vanderbilt University, Nashville, TN, United States; 3 Department of Biomedical Engineering, Vanderbilt University, Nashville, TN, United States; 4 Department of Chemistry, Vanderbilt University, Nashville, TN, United States; 5 Department of Chemistry and Physics, Belmont University, Nashville, TN, United States; 6 Department of Biomedical Engineering, University of Iowa City, Iowa, IA, United States; 7 Department of Medicine, Division of Nephrology, Vanderbilt University Medical Center, Nashville, TN, United States; 8 Department of Pharmacology, Institute of Chemical Biology, Center for Applied Artificial Intelligence in Protein Dynamics, Vanderbilt University, Nashville, TN, United States; 9 Institute for Drug Discovery, Institute for Computer Science, Wilhelm Ostwald Institute for Physical and Theoretical Chemistry, University Leipzig, Leipzig, Germany; 10 Center for Scalable Data Analytics and Artificial Intelligence ScaDS.AI and School of Embedded Composite Artificial Intelligence SECAI, Leipzig, Germany

**Keywords:** epitope-focused vaccine design, glycan masking, glycan shield, glycoengineering, immunogen design, N-linked glycosylation, protein-glycan modeling, reverse vaccinology

## Abstract

Epitope-focused vaccine design aims to improve upon existing immunization strategies by eliciting immune responses against specific epitopes targeted by known therapeutic antibodies. One of the techniques in epitope-focused immunogen design is glycan masking, in which sugars are used to hide epitopes on the protein of interest that are associated with low therapeutic potency. Here, we provide a detailed overview of the computational and experimental techniques associated with glycan masking for immunogen design at a biochemical and biophysical level. We will cover well-established and emerging *in silico* methods for predicting and engineering glycosylation sites. Additionally, we will discuss expression and validation of glycosylated immunogens *in vitro*. We hope this review will be a useful overview for scientists interested in performing glycan masking in their field of research.

## 1 Introduction

Glycan masking is a natural phenomenon in which viruses escape the immune response by masking therapeutic epitopes with poorly reactive sugars (1–3). This phenomenon is very common among viruses and their envelope glycoproteins, which are the first target of an antibody-mediated immune response. As B-cells maturate and their elicited antibodies’ specificities are improved, the newfound selective pressure causes viruses to evolve towards escaping recognition by immune cells. In particular, influenza hemagglutinin (HA), HIV envelope (Env) and coronavirus Spike (S), are often heavily glycosylated and can acquire additional glycosylation sites through evolution (4–7). Glycans can prevent antibody binding through steric hindrance, by physically blocking access to the target epitope. Based on natural viral evasion mechanisms, researchers have developed glycan masking techniques as novel methods in vaccinology, redirecting the immune response aimed at specific outcomes (8).

**Figure 1:**
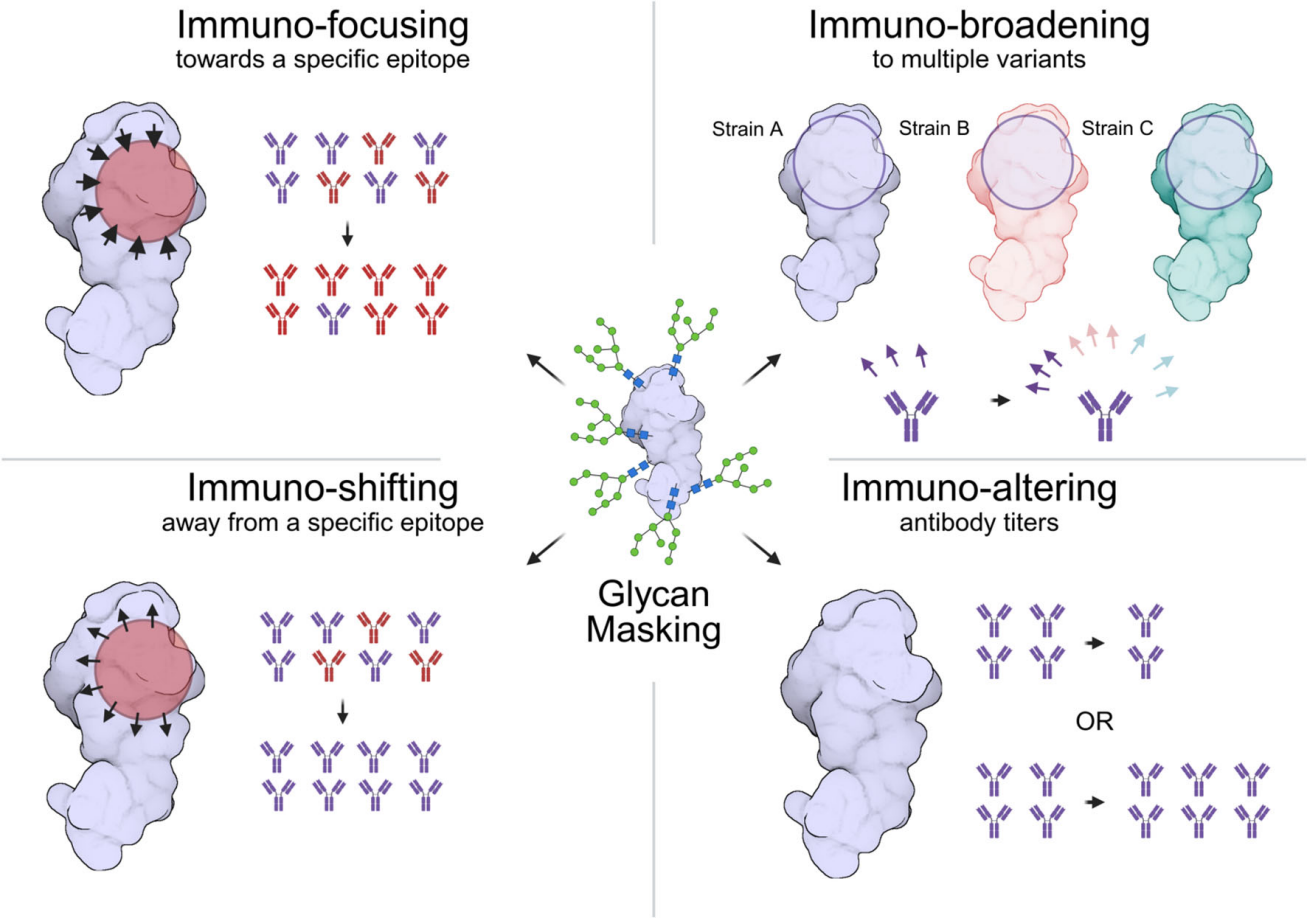
Application of the glycan masking technique in immunogen design: focusing, shifting, broadening and altering. The glycosylated immunogen is represented in violet, while alternative strains are shown in pink and green. Epitopes of interest for focusing and shifting are shown in red, and the antibody immune response is color-coded to match the epitope of interest. Created in BioRender. Martina, C (2026). https://BioRender.com/bhxzsus.

In epitope-focused vaccine design, glycan masking can, for example, be used to immuno-focus the antibody-mediated immune response to epitopes that elicit therapeutically relevant antibodies (Abs), such as neutralizing antibodies (nAbs), or broadly neutralizing antibodies (bnAbs, see **Figure 1**). nAbs are Abs that can neutralize pathogenic infection by binding, and can do so through a variety of mechanisms, such as blocking viral receptor binding sites (9). bnAbs are nAbs that can neutralize a wide range of pathogenic strains, generally by binding to epitopes with high sequence and/or structural similarity between pathogenic strains. It is important to note that neutralization is not always necessary for an antibody to confer protection against a pathogen-of-interest (10, 11). In glycan masking, glycosylation sites are engineered such that irrelevant epitopes are blocked by glycans, allowing, e.g. bnAb epitopes, to be preferentially recognized. Glycan masking has been applied to multiple pathogens, including HIV (12–17), influenza virus (18–20), Zika and Dengue virus (21) (for more information and examples, see Martina et al. (8)). Alternatively, glycan masking can be used to immuno-shift the response away from hypervariable, immunodominant epitopes and/or artificial regions (e.g, scaffold proteins like nanoparticle platforms, non-native residues introduced during antigen engineering, etc.) which would not elicit a broad or potent response (see **Figure 1**). This has also been performed for HIV (22, 23), influenza virus (18, 20, 24–28), malaria (29), and some artificial scaffolds (30–32). Finally, among the most common applications, glycan masking can also be used to immuno-broaden the response by eliciting cross-reactive antibodies (12–16, 18–20, 24, 25, 28, 33, 34) and to immuno-alter the response by lowering or increasing the overall antibody titers (17, 29, 31, 32) (see **Figure 1**).

Glycan masking is a very efficient way to redirect the immune response because the *N*-glycans presented by viruses (i.e. GlcNAc) are poorly reactive (35), with only a few classes of antibodies known to bind to them (36–38). Given that glycosylation is one of the most common post-translational modifications in human proteins (39), antibodies may have evolved to not recognize “self” sugars produced by the host cells. By replicating within human cells, viruses adopt a human-like pattern of glycosylation and sugar propensity, thus being ignored by the immune system antibodies (35).

Glycan masking uses *N*-linked glycosylation, in which a nitrogen atom on an asparagine connects the protein and the sugar moieties, as opposed to O-linked glycosylation, which uses an oxygen atom (40, 41). In *N*-linked glycosylation, the amino acid sequon (NxS/T) recognized by glycosyltransferase enzymes is necessary for the addition of the first sugar ring (42) to the asparagine, while the second residue can be any amino acid except proline and the third residue can be either serine or threonine.

Engineering novel glycans into proteins is simple from a theoretical standpoint, but problems in protein expression and folding might arise (43, 44). However, well-placed glycosylation can help in increasing the solubility and overall stability of the proteins of interest (45, 46). These different outcomes are often protein-specific and position-specific, and it is challenging to identify viable, novel glycosylation sites on a protein. Various *in silico* tools have been developed over the years to help researchers with glycan modeling and predictions. Similarly, experimental methods to express and validate glycoproteins are being refined and innovated. Given the importance of glycan masking in the development of biotherapeutics such as vaccines, understanding the array of available tools is of extreme importance.

In this review, we will highlight established procedures, alternative approaches, and emerging technologies for glycan masking in immunogen design. We report currently available tools for predicting glycosylation propensity at the residue level, and for modeling glycans onto the protein of interest. We will include both recent methods that leverage artificial intelligence (AI) and classical non-AI methods. We also provide an overview of standard and novel methods that are used in glycan masking of immunogens, including protein expression, glycan presence and validation of protein folding. We hope this review will work as a guide for researchers working with glycans and glycan masking in immunogen design and beyond.

## 2 Computational techniques: predictions

Initial efforts in immunogen design through glycan masking aimed to predict positions in antigens that were most suitable for glycan insertion. Two main techniques have been used: alignment to homologous sequences, and sequence predictors. Each technique will be described in the following sections, but a summary of publications using these techniques is reported in **Table 1**. Additionally, a detailed overview of the historical development of AI tools in glycobiology, known as glycoinformatics, has been published by Bojar and Lisacek (47). In particular, datasets in glycomics, glycoproteomics, and glycan-binding have been used for training of various tools, from glycan classifiers to predictors for glycosylation.

**Table 1:** List of publications using computational techniques to predict sites for glycosylation and to model glycans.

Technique	Publication	Details
Alignment of homologous sequences	Lin SC. et al., 2012 (24)	163 different sequences of influenza H5 HA
	Bajic G. et al., 2019 (18)	Available sequences for influenza H3 HA
	Boyoglu-Barnum S. et al., 2020 (19)	Available sequences for influenza group 1 and 2 HA
Predictors	Duan H. et al., 2018 (17)	NetNGlyc
Sequon modeling	Sampath S. et al., 2013 (29)	Rosetta
	Ingale J. et al., 2014 (16)	Rosetta
	Duan H. et al., 2018 (17)	Pymol
	Adolf-Bryfogle J. et al., 2024 (32)	Rosetta
Glycan modeling	Ingale J. et al., 2014 (16)	–
	Duan H. et al., 2018 (17)	Glycosylate
	Adolf-Bryfogle J. et al., 2024 (32)	Rosetta
	Lemmin et al., 2019 (55)	Python
	Krishna et al., 2024 (54)	RoseTTA Fold All Atom
	Abramson et al., 2024 (53)	AlphaFold3
	Ives et al., 2024 (63)	GlycoShape
	Dialpuri et al., 2024 (84)	Privateer
Molecular Dynamics	Ingale J. et al., 2014 (16)	–

The first column reports the technique name, the second column reports the publication references and the third column reports additional details.

### 2.1 Alignment to homologous sequences

Early efforts in identifying putative glycosylation sites for immunogen design were done by analyzing homologous sequences of viral proteins. For example, Lin SC. et al. (24) analyzed the sequences of 163 highly pathogenic influenza A H5N1 strains identified in human samples to identify the most variable regions, which were consequently masked through glycosylation. The rationale behind masking hyper-variable regions is to expand the breadth of action of the vaccine and to make it suitable for preventing infection from many H5N1 strains simultaneously. A total of nine glycosylation sites were identified from five hypervariable regions, but only six of them presented the engineered glycosylation upon SDS-PAGE inspection.

Similarly, Bajic et al. (18) aligned all available sequences of influenza H3 from the 1968 Hong Kong outbreak (H3 HK68) and saw that during viral evolution the number of glycosylation sites in HA doubled over time from 7 to 14. They then designed three hyperglycosylated immunogens starting from H3 HK68. In the first mutant, five additional glycosylation sites were added to mask everything except for the receptor binding site (RBS) epitope, totaling 12 glycans. The second presented the same five additional glycosylation sites as the first mutant, in addition to a glycosylation to conceal the RBS, for a total of 13. The third presented four additional glycans on top of the second, for a total of 17 glycans potentially able to mask the full surface of the protein. Following mouse immunizations, the authors were able to show that the additional engineered glycans do not affect the overall antibody titers and that the sera from each sample was able to recognize the WT protein H3 HK68. However, the different glycovariants elicited different classes of antibodies, enriching in specific germline V_H_ genes 1-69, 1-9, and 5-9-1 (18). This shows that glycan masking can enhance frequencies of preferred V_H_-genes and that glycosylated immunogens can contribute to germline targeting.

**Figure 2:**
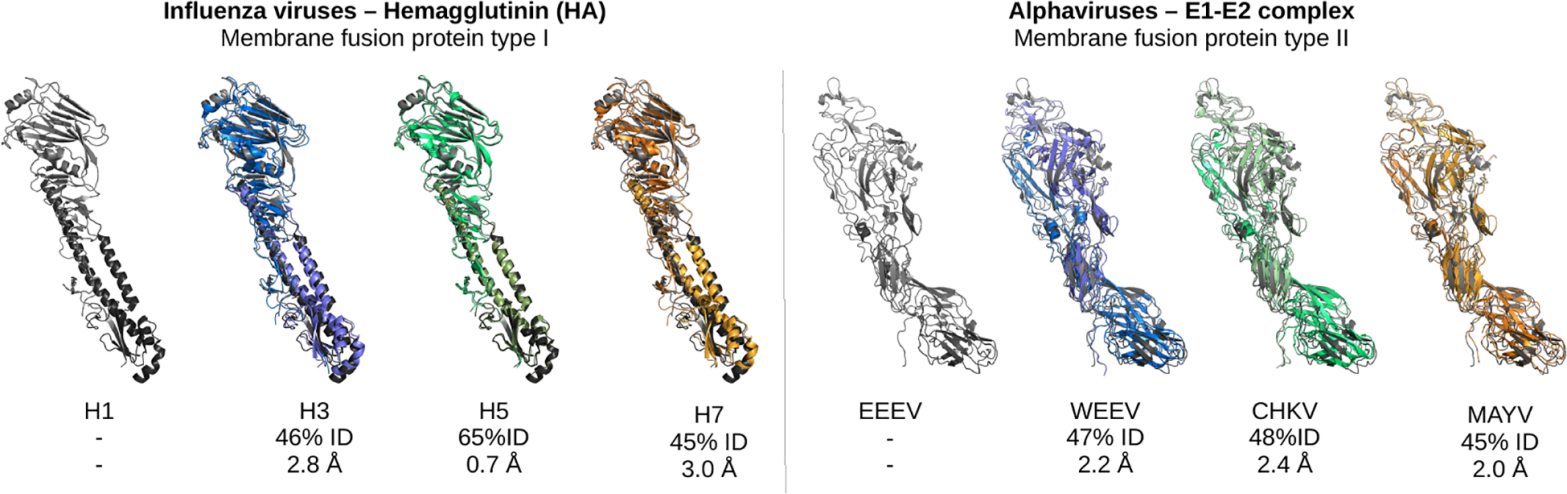
Sequence and structural similarities of viral membrane fusion glycoproteins type I and type II. Representation of the influenza hemagglutinin protein (left) and the alphavirus E1-E2 complex (right). In particular, for influenza virus a one-to-one comparison of H3N2 is shown in blue (A/Hong Kong/1/1968, pdb ID 6NHR), H5N1 in green (A/Indonesia/5/2005, 4K67) and H7N9 in orange (A/Shanghai/02/2013, 5VJK) against H1N1 in gray (A/Brevig Mission/1/1918, 4GXX). For alphaviruses, WEEV is shown in blue (PDB ID: 8DAQ), CHKV in green (8FCG), MAYV in orange (7KO8) against EEEV in gray (6XOB). The reported sequence identities (percentages) were obtained from Blast and the RMSD values were calculated with Pymol.

The identification of potential glycosylation sites through sequence alignment is a successful strategy due to the high structural similarity of the proteins within viral families. For example, HA of influenza virus H1 from 1918, which is a type I membrane fusion protein, bears a sequence identity of 46% to H3 HK68, 65% to an H5 strain from 2005, and 45% to an H7 strain from 2013. However, the structural similarity of the HA1-HA2 complex is 2.8 Å to H3, 0.7 Å to H5 and 3.0 Å to H7. Similarly, type II membrane fusion glycoproteins such as those of alphaviruses have low sequence similarity between different species (below 50%), but high structural similarity (see **Figure 2**). Structural conservation is generally more relevant than sequence similarity since glycans need to be solvent accessible and fully exposed on the surface of the protein. Sequences might vary substantially but still encode for the same fold, and may thus be misleading. The high structural similarity of viral proteins suggests that a potential glycosylation site present in one strain may be well-tolerated in the same structural region of another strain. Thus, this simpler method of selection for glycosylation sites could be highly effective.

### 2.2 Glycosylation predictors

Glycosylation predictors are computational tools to identify and predict sites on the protein of interest that are most likely to undergo natural or engineered glycosylation. An overview of the development and principles behind various glycosylation predictors are given below.

Different generations of glycosylation predictors have been developed over time (47). The predictors from various generations mainly differ from two aspects, one is the feature extraction input, which can be sequence based only or sequence/structure hybrid. The other is the algorithm underneath the tool, traditional machine learning algorithms such as tree forest, support vector machine (SVM) and neural networks are popular options at early generations, but the usage of more advanced algorithms such as protein language model (PLM) becomes more popular since its release around 2023. The first generation, available before 2005, includes bioinformatic tools to identify the presence of the NxS/T sequon within the amino acid sequence of the protein of interest and to evaluate its propensity for glycosylation. An example of such sequence-based tools is NetNGlyc (48), which was used by Duan et al. (17) to identify 13 novel glycosylation sites to insert in an anti-HIV nanoparticle immunogen named eOD-GT8 60-mer, which already presents ten native glycosylation sites. Duan and team (17) designed 13 glycovariants by adding a single glycosylation to the wild-type eOD-GT8 60-mer, and 35 additional variants presenting two glycans simultaneously. All 48 constructs successfully expressed in mammalian cells, and most retained binding to the epitope of interest (CD4 binding site) by target antibodies. The single variants, however, performed worse in masking non-CD4 epitopes, showing that the presence of multiple glycans was more efficient in masking irrelevant epitopes.

Later generations of predictors included additional information such as structural, functional and evolutionary features. Tools like GlycoMine (49, 50) and N-GlyDE (51) have been widely used to glycosylate viral proteins, though they have rarely been used to identify potential sites for immunogen design through glycan masking. Potential reasons include the fact that the tools were trained only on natural sequences, and that datasets of engineered glycovariants, which could improve the model training, are currently missing or too small. In order to utilize these tools in an immunogen design context, one can apply them to screen engineered immunogens for glycosylation sites. By evaluating the probability of glycosylation at a given site or introduction of glycosylation in the design process, researchers can filter designs that remain unoccupied to increase the chances of successfully masking the target area on the immunogen and ensuring that unwanted epitopes are properly shielded.

## 3 Computational techniques: modeling

With recent advances in structural biology and protein modeling, multiple *in silico* tools have allowed for the accurate modeling of glycans on viral glycoproteins and on immunogens with varying accuracy and speeds. Different tools now allow glycosylation of proteins via sequon introduction, modeling of linked glycans, and evaluation of overall stability. In this section, physics-based and AI-based methods of glycoprotein modeling will be discussed, as well as their applications to the field of glycan masking and immunogen design. The most common techniques in epitope-focused immunogen design are summarized in **Table 1**.

In general terms, the modeling process typically begins with preparation of the target protein, ensuring sequons are present. Then, relevant glycans are selected for addition to the target protein, and finally the model is built. These tools for glycan modeling have been often utilized for immuno-focusing, increasing chances of success before *in vitro* assessment and playing an important role in vaccine design. Tools used for this application include Rosetta (52) and other downloadable software suites, in addition to several web-based platforms. AI-based tools like AlphaFold3 (53) or RoseTTAFold All-Atom (RFAA) (54), each with unique algorithms and user interface, have been developed to also model glycans and other small molecules. All tools introduced in this section are summarized in **Table 2**, although some of them are no longer actively maintained or accessible. Still, their contributions to the field are worth mentioning.

**Table 2:** Comparison of computational tools for glycan-protein modeling.

Name	Cost	User interface	Algorithm	Evaluation metrics	Input file	Time efficiency	Type
Rosetta	Free for academia	Terminal	Monte Carlo optimization	Rosetta energy score (REU)	pdb	Slow	Physics-based
Glycosylator	Free	Terminal or GUI	CHARMM	N/A	pdb	fast	Physics-based
Glycoshape	Free	GUI	Steric optimized placement algorithm	N/A	pdb	fast	Physics-based
CHARMM	Free for academia	Terminal or GUI	MD	N/A	pdb	slow	Physics-based
RF All-Atom	Free for academia	Terminal	Deep learning	Confidence score, pLDDT and others	Fasta + SMILES	Normally faster than Rosetta	AI-based
AlphaFold3	Free for non-commercial	Terminal or GUI	Deep learning	Confidence score, pLDDT and others	Fasta + CCD codes	Similar to RF all- atom	AI-based
Privateer	Free	GUI	Cremer-Pople algorithm	Conformations, Torsions and others	pdb	fast	Other

Six aspects are taken into account: cost, user interface, underlying algorithm, evaluation metrics, input file type, time efficiency and type. N/A, Not applicable.

### 3.1 Physics-based glycan modeling

Four commonly used software platforms perform physics-based glycan modeling: Rosetta Suite (32, 52), Glycosylator (55) GlycoShape and Molecular Dynamics (MD) simulation. The Rosetta software suite has played a significant role in general protein modeling and predictions, with the carbohydrate framework integration allowing computational glycan modeling and structural assessment (32, 56, 57). Specifically, the GlycanTreeModeler tool allows for the *de novo* glycosylation of input constructs at designated glycosylation sites; this is not, however, without limitations. Due to the extensive sampling needed to assess the glycans’ rotational degrees of freedom; the approach can be computationally expensive. Additionally, current protocols use defined glycoforms, and this can lead to difficulty in predicting glycan microheterogeneity. Finally Rosetta can be sensitive to starting structures potentially affecting the final result, where certain backbone coordinates or glycan orientations may lead to traps in local energy minima. The protocol behind GlycanTreeModeler mimics the growth of trees, and the sugar root linked to the protein is modeled first and energy minimized, then the second layer of sugars are modeled and energy minimized and so on until the last sugar. The protocol uses Monte Carlo optimization and sampling of the glycans’ freedom of movement (GlycanSampler). To do this, the algorithm assesses torsion angles, side chain optimization, and evaluates the optimization of the glycosidic linkages as it builds, and scores the resulting structures through a Rosetta-specific scoring function. The result is a glycosylated protein with a conformation similar to that of experimentally-determined structures (32). While it is true that the resulting glycoproteins are in agreement with experiments, we must note that the conformations predicted by Rosetta are approximations. It is also important to note that *in vivo* dynamics are significantly more flexible and present a more stochastic nature than those predicted or sampled by Rosetta.

Recently, GlycanTreeModeler has seen use in the glycan masking space to lower the overall immunogenicity of a scaffold protein. In a 2022 study by Kraft et al. (58), protein nanoparticles presenting HIV-based immunogens were glycosylated to shield them from the immune system and allowing the antigen to remain immunodominant over the nanoparticle scaffold. This allowed for the antigen to remain immunodominant over the nanoparticle scaffold. In that study, they evaluated the tertiary structure of the original nanoparticle, looking for outward-facing residues which were optimal for sequon placement. They then used CreateGlycanSequon mover for sequon insertion, and GlycanTreeModeler for glycosylation at the desired site. Finally, the designs were evaluated *in vivo.* There, they found that if the epitope is immuno-dominant to the scaffold being used, which is accomplished via glycosylation, there is no hindrance to the immunogenic effect of the antigen (58). Additionally, in a 2023 study by Dosey et al. (59) it was found that hyperglycosylation of stabilized immunogen candidates both increases and focuses antibody-mediated response to the target epitope in rabbits. In the creation of these glycosylated constructs, Rosetta was used to both guide sequon placement and in the modeling of the glycans before moving to *in vitro* and *in vivo* experiments.

Glycosylator is a Python-based program designed to model glycosylation via flexible interaction modes, including direct execution at the command line, in a Python script, or via the affiliated graphical user interface (GUI) (55). Contributing to the platform’s ease of use and flexibility, Glycosylator has also been added to the BuildAMol toolkit which combines multiple programs made in Python for the purpose of molecular modeling (60). Glycosylator allows users to specify their protein of interest and the positions in which they would like glycosylation; there is also a sequon finder that can glycosylate automatically. Functionally, the platform focuses on the structural placement of glycans with users being able to determine glycan placement based on the integrated sequon finder. The user can either provide a PDB file of the glycan and the protein, or they can create the glycan directly using the glycan’s IUPAC name. When written into the script, Glycosylator outputs the glycan that can then be used for glycosylation. The program subsequently samples multiple conformations of the sugars and their subsequent glycosylations, resolving any structural conflicts or clashes that are found and sampling geometrically viable conformations. While the program does resolve clashes, before using the structure in platforms such as Rosetta for assessment, it is recommended that the structure be refined first. Glycosylator’s functionality can be useful in the field of immunogen design, where modeling glycan shielding can aid in predicting blocked sites. Additionally, the program can be used to inform vaccine design by aiding in the understanding of conformational states of the target protein, and the effect that this can have on antibody binding. More specifically, the conformational sampling can allow for the prediction of potential steric clashes between the introduced glycan shield and the effect this may have on interaction between the antibody and the immunogen. This can be useful in confirming that engineered glycans effectively shield other potential epitopes without accidentally occluding the desired interaction site. Additionally, Glycosylator can be used in the addition of glycans for locking viral proteins in a prefusion state, as is often done in immunogen design against pathogens like HIV. For example, Bennett and colleagues (61) show, via microsecond-long MD simulation, that glycosylation at areas of interest can be used to lock the envelope protein of HIV-1 in the prefusion state, which has implications relevant to vaccine design due to the conformational changes that are needed prior to host cell entry. Additionally, Glycosylator has seen use in increasing the understanding of antibody antigen interactions through ascertaining the binding distance between antibody and antigen (62). In a 2021 study by Lee et al. (62), the authors note that the glycosylation of epitopes targeted by antibodies against HIV-1 increases the antibody-antigen distance in binding, while the creation of glycan holes decreases this distance. Glycosylator was utilized in the creation of glycosylated structures for subsequent MD evaluation, displaying that the program can be used to study natural phenomena. However, Glycosylator can also be used for engineered constructs, which can be studied through MD or other methods like protein docking. Although the examples do not directly address the design of immunogens, Lee et al. provide an example of how glycan addition or absence can alter the dynamics of protein-protein interactions, specifically antibody-antigen interactions, which is of great interest to those designing immunogens. Additionally, in the work by Bennett et al., Glycosylator is used to alter the protein to study the prefusion state of the envelope protein of HIV-1. Here, glycan alteration displays changes in the conformational state of a viral protein by biasing one state over another. In immunogen design, especially when there is a state of interest, like a prefusion state, design may be done to favor this state over another using programs such as Glycosylator.

GlycoShape is a free computational platform designed to build and visualize glycoprotein structures. The input structures can be derived from existing PDB files or AlphaFold predictions (63). This platform addresses the challenge of missing or incomplete glycan representations in experimental or predicted protein models by adding realistic glycan conformers using a steric-optimized placement algorithm. This algorithm evaluates whether glycans are compatible with protein surface by selecting glycan conformations from a curated library that minimizes steric clashes while maintaining biologically plausible orientations. The platform has a user-friendly web interface. For input, the user uploads a target protein structure in PDB format, then selects a glycan from its dropdown glycan list. After submitting the job, users can directly visualize the resulting glycoprotein structure within the browser, without installing another protein structure visualization software. This visualization is powered by WebGL (Web Graphics Library) and supports output download in standard formats (e.g., PDB) for use in downstream applications or simulations. GlycoShape also supports an “Advanced Glycosylation” mode, by applying it users are able to model multiple glycosylation sites simultaneously (typically 5–10 glycans per run). However, modeling many glycan sites at once can increase processing time and potentially introduce steric clashes, depending on protein surface topology. Although the platform is very user-friendly, a notable limitation of GlycoShape is its absence of quantitative scoring metrics to evaluate and compare the energy landscape or structure quality of the generated glycoprotein models. Therefore, users who would like to have evaluation metrics of the models may need to feed GlycoShape output structures into other modeling tools like Rosetta or run MD simulations for further assessment. This platform has seen use in stabilization of HIV-1 Env, specifically being used to predict the areas which may be occluded by glycosylation and therefore masked from immune response (64). Kumar and colleagues (64) used multiple tools for glycosylation including GlycoShape emphasizing an approach in which multiple prediction tools can be used to see if a consensus is reached amongst them.

MD simulations allow for the investigation of molecular systems at the atomic level (65). While static structural models have the limitation of oftentimes underestimating the coverage of glycan shielding and potentially missing transient occlusion events, MD allows for the capture of the full dynamic range of the glycan shield and an understanding of these events, if present. Due to the great conformational diversity that glycans display, MD simulations can aid in the representation of the conformational landscape of glycoproteins, thereby allowing researchers to better understand the scope of glycan masking. For example, in a publication by Re and Mitzuguchi (66), MD was employed to assess the glycan shield dynamics of Lassa virus to assess where glycan shielding may affect binding of antibodies, stating that this work may aid and influence vaccine design efforts in deciding which antibody response or epitope target sites on the virus are optimal for targeting based on occlusion caused by glycan shield dynamics (66). While MD provides these benefits, some of these phenomena occur on microsecond to millisecond timescales, which can be prohibitively computationally expensive to simulate, depending on the available resources. This idea of increasing our understanding of glycan conformational heterogeneity through computation has aided in our understanding of how viruses use glycans dynamically, and in the design of immunogens. In selecting a glycoprotein modeling platform, researchers may be choosing between Rosetta and Glycosylator. It is important to mention that these two platforms do not necessarily have the same depth and usability, and are not necessarily used for the same purpose. For example, Rosetta offers a comprehensive scoring function for assessing structures and more rigorous structural refinement, while Glycosylator offers a more accessible interface and rapid structure generation. Rosetta also offers more options for assessment methods and potentially for structure filtering when used in a protein design context, whereas in Glycosylator, multiple platforms might be needed for design and filtering of immunogen designs. Both programs are useful in the field of immunogen design, and the decision of which to use is ultimately up to the user and largely depends on the computational rigor of their experiments and the computational resources available.

Accurate MD simulations involve the consideration of several simulation conditions, including the forcefield (67). Force fields are used to calculate the potential energy landscape of the system as a function of particle coordinates. There are force fields that have been made specifically with consideration paid to the dynamics of glycans, such as GLYCAM/AMBER force fields (68–70), and there others, such as CHARMM have added the functionality to assess glycoproteins over time (71). Hybrid approaches combining protein and glycan simulation methods, including AMBER and GLYCAM, are commonly used and widely validated for viral glycoproteins. It is important to emphasize that oftentimes the reliability and accuracy of MD simulations can depend on the force field chosen, and there has been much effort paid to comparing force fields for both glycoproteins and protein-carbohydrate complexes (72). In addition to the force field, great attention is paid to many other factors such as the simulation box, solvent, temperature, pressure, equilibration steps and other simulation conditions based on the question being answered.

In addition to viral immune escape, glycans can play a large role in the host cell entry of viruses. It has been found via MD simulation and subsequent *in vitro* experiments, that hinge glycans on the spike protein of some coronaviruses increase infectivity and aid in cell entry (73). Studies investigating SARS-CoV-2 and its glycan shield have found regions that could be susceptible to human leukocyte antigen detection due to gaps in glycosylation (74). In addition to the study of naturally occurring glycan shields, MD simulations can aid in the design of immunogens. For example, the glycoprotein prediction platform GlycoShape, utilizes individual MD trajectories, that they congregate into a single dataset allowing for the prediction of a conformational landscape (63). Platforms such as GlycoShape allow for the accurate prediction of not only single structures but also consider the structural diversity of glycans, which plays a large functional role in entry, protection from the immune system, and now in the design of immunogens utilizing glycans to expose epitopes of immunological relevance. For example, Nuqui et al. (75) successfully created simulation-informed immunogen designs for SARS-CoV-2 using information on dynamics, specifically the breathing of the S2 trimer, that was not directly visible in Cryo-EM determined structures alone. The MD simulations conducted here informed the substitution of tryptophans used to lock the S2 trimer in the perfusion state, creating a protein that was more thermostable and elicited an antibody response in mice (75).

### 3.2 AI-based approaches to glycosylation

In addition to the aforementioned downloadable software packages for the modeling of glycoproteins and the assessment of glycan conformations there are also several web-based and AI platforms that seek to improve glycan modeling, such as RFAA (54), AlphaFold (53, 76), GlycoShape, and others described below. These types of tools, broadly speaking, have pros and cons that should be considered before modeling or creating a pipeline for the modeling of glycoproteins. For example, these tools often have differences in their time efficiency, as the generation time per structure, when compared. Rosetta typically takes the longest, especially if the defined protocol involves an energy minimization step, which is typical for many modeling pipelines (52). For example, for a 2300 aa amino acid long sequence modeled with mannose-5, the time needed can be around one hour (32). The AI based platforms, such as AlphaFold (53, 76) or ColabFold (77), are typically quicker and can take as little as a few minutes for the generation of a structure (78, 79). For context regarding the general needs of each platform type and their pros and cons, refer to **Table 2**.

RFAA is a structural prediction network that allows for the *de novo* structural modeling of proteins as well as small molecule, nucleic acid, and glycan spatial prediction with an input FASTA sequence and IUPAC glycan name for glycoprotein modeling (54). For the accurate prediction of structures, RFAA uses a three-track neural network utilizing a 1D sequence alignment track, a 2D distance matrix, and a 3D coordinate matrix, while taking into account the sequence, residue distances, and positions, respectively. The use of deep learning platforms for the prediction and/or modeling of glycans in immunogen design, is fairly new, with RFAA’s publication in March of 2024. Specifically in the glycan masking space for vaccine design, biophysical methods still outperform AI-based methods. Because of this, there have not been many applications to immunogen design and glycan masking. However, given the promise of RFAA and other similar deep learning platforms, the accuracy of glycan masking predictions will likely be increased, and the protocols for the modeling of a glycoprotein will be simplified over time, allowing a larger range of researchers to adopt the technology for this purpose. It is worth noting that most ML-based structure prediction platforms, like RFAA and AlphaFold2, were designed to yield ‘the best’ structure given a protein sequence. Confidence metrics enable these algorithms to rank the quality of each structure. This is useful for traditional applications, but is not as useful when an ensemble of protein structures is needed, as in determining the potential space occupied by a glycan chain. These tools can be modified to yield ensembles of protein structures, but may be more computationally expensive than, for example, traditional approaches like Rosetta or MD (80). Ensemble representation of glycans can be beneficial as they possess significant rotational freedom and therefore, do not occupy a fixed location. As a consequence, the use of ensemble representation is important in the determination of the occupancy of the glycan shield, in terms of probabilistic location of the glycans. This allows researchers to better distinguish between constantly shielded epitopes and those which may be transiently revealed. On the other hand, while the ensemble-based approach to glycoprotein modeling offers a realistic representation of biological reality, it can introduce computational cost, as previously mentioned.

AlphaFold3 (AF3) is an AI-based protein structure prediction tool developed by DeepMind (53). Compared to its previous version AlphaFold2 (AF2) (76), and AlphaFold Multimer (AFm) (81), key improvements were made to support a broader range of molecular species. While AF2 and AFm can only predict protein structures or complexes, AF3 can also predict interactions of proteins with other molecule types, including DNA, RNA, small-molecule ligands, and glycans. This expanded functionality allows AF3 to model glycoprotein structures. To use AF3 to model glycoproteins, users provide the protein’s amino acid sequence along with Chemical Component Dictionary codes (CCDs) (82) representation of the glycan, rather than providing pre-existing structural files like PDBs. AF3 provides scoring metrics to evaluate the reliability of its predictions. These metrics include the confidence score (ranging from 0 to 100) and pLDDT (predicted Local Distance Difference Test, ranging from 0 to 100). Higher scores indicate higher confidence in the reliability of the predicted structure. Confidence values above 70 often are considered reliable, and values above 90 indicate a very high confidence in structural accuracy (83).

### 3.3 Others - tools for assessing glycoprotein models

Unlike other tools described in previous sections, Privateer is not a tool for generating glycoprotein models, but a tool that provides validation metrics for existing glycoprotein structures (84, 85). According to its website, Privateer doesn’t model glycoproteins directly but provides a quality assessment of modeled or experimentally determined glycans. It extracts sugar molecules from the input PDB structure by user, then calculates sugar molecules’ parameters according to the Cremer-Pople puckering metrics (86) to confirm their conformations, then compares them to statistically derived distributions from high-quality PDB structures to evaluate how plausible the conformations of the extracted sugar molecules are. Finally, it gives a validation report to inform the user of any potential issues the glycans may have in the given glycoprotein. For example, the report includes fields like number of conformation issues, number of torsion issues and so on.

Finally, GLYCO (87) is another program used for the assessment of glycan masking, while it does not glycosylate directly, it does assess glycan sterics of previously predicted structures. This can inform immunogen design in ascertaining the needed number of glycosylation sites to enhance immunofocusing, in addition to ensuring that glycans do not occlude the needed epitope.

### 3.4 Glycan masking with physics-based versus AI platforms

When considering the AI and web-based protocols for the modeling of glycoproteins (54, 63, 76, 77, 81), it is important to consider the differences in how they model when compared to platforms such as Rosetta that utilize a more physics-based approach comparatively (32, 52, 56). Rosetta utilizes predefined energy functions and scoring terms to assess glycan conformations and generally is useful for the generation of high-resolution structures and quantitative scoring metrics. Rosetta also allows the exploration of the conformational space via the creation of an ensemble of structures rather than single best conformation, as is typical for AI-based platforms. This sampling of the conformational space can aid in distinguishing areas of the immunogen accessible to antibodies and those that are not. Additionally, Rosetta and other platforms like it, allow for customizability in the glycans being added, so the more niche parts of the field using novel or less common glycan structures may be supported in Rosetta but not on AI platforms, although this is changing as the field progresses. Due to this high resolution assessment of the conformational space however, Rosetta, as previously mentioned, takes more time and has a steeper learning curve when compared to tools like AF3, so if quicker results are needed then AI-based platforms should be considered. Additionally, AI-based platforms allow for the folding of proteins *de novo* with the user providing a sequence and glycosylation site. This allows for the aforementioned combined approach, which is common in the field of using tools like AlphaFold3 for protein structural prediction and Rosetta for glycosylation as a part of a glycoprotein modeling pipeline.

The modeling of glycans onto proteins is complex and requires intensive biophysical analysis, and the rise of AI and web-based protocols in addition to Rosetta and other physics-based systems is becoming more common also in specific fields such as glycan masking for immunogen design. The use of these programs in conjunction, in particular performing protein modeling with AlphaFold and glycan design with Rosetta, is currently the preferred method in glycan masking for immunogen design, and it combines the best of both fields.

## 4 Experimental techniques - glycoprotein biosynthesis

**Figure 3:**
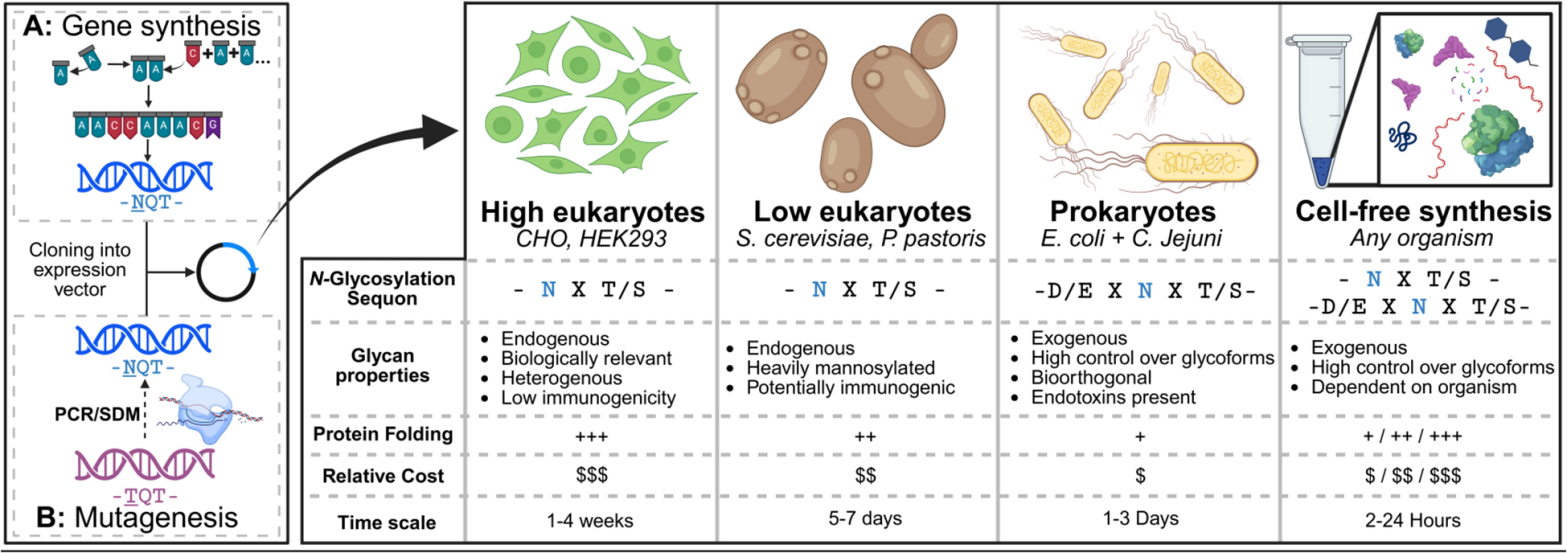
Approaches to glycan-masked immunogen biosynthesis. The left panel illustrates the two main routes to acquire a glycan-masked mutant, gene synthesis and mutagenesis. The right table highlights some of the most popular routes to produce glycoprotein immunogens, and some considerations for choosing an expression system. Symbols (+ and $) are relative and arbitrary, but are based on reported analyses of costs between the expression systems (92, 153, 212, 213). Protein folding describes the likelihood of successful soluble production of large and/or complex proteins. CFPS cost and folding capacity depends highly on the organism used, and biochemical parameters set by the user (214). Time scale refers to harvesting time after DNA is introduced to the expression organism/system. Note that these parameters may deviate drastically depending on the protein. Created in BioRender. Araiza, G (2026). https://BioRender.com/dr563ow.

In many cases, the presence of N-linked glycans can improve biophysical and biochemical characteristics such as solubility, proteolytic stability, thermostability, half-life, and overall yields, provided the glycan does not interfere with the protein’s folding or function (41, 44). Virus-associated immunogens in particular are usually robust to glycosylation along solvent-exposed regions and loops (59, 88, 89). Some viral proteins, such as HIV Env, are even hyperglycosylated and can contain 30 or more N-linked glycans on its gp120 subunit alone. However, not all pathogen-associated proteins can readily support artificially introduced glycans, and addition of glycans may even result in adverse immunogenic reactions (90). For synthesis of glycoprotein immunogens, the preferred route in the relevant literature is overwhelmingly in favor of eukaryotic cellular expression systems, primarily those of human cell lineages (91). These systems are robust and usually do not require highly specialized cell lines or host genome modification, though this may be a case-by-case consideration. Here, we discuss general strategies for the biosynthesis of designed glycoprotein immunogens. We will highlight systems that are commonly used and offer alternatives that may be more accessible or convenient to researchers (**Figure 3**).

### 4.1 DNA, genes, and vectors

The first consideration scientists must take in validating their designs is how they will acquire the genotypes encoding their designed glycoimmunogens. DNA expression vectors are circular DNA that encode the information necessary for an organism to produce a protein exogenously. DNA vectors are typically between 3,000 to 10,000 base pairs (bp), though some vectors can be larger than 30,000 bp (92). The variance in size stems from the modularity of vectors and their features, as they encode the biological information needed for the organism to replicate the DNA, express the protein, traffic the protein, and so on. In addition, vectors can also encode features desirable to researchers, such as selection markers, purification tags, and so on (92, 93). The most direct approach for acquiring artificial genes is through oligonucleotide synthesis, for which many third-party services exist (94). These genes can then each be cloned into an expression vector. Advances in oligonucleotide synthesis have made acquisition of novel DNA sequences highly affordable and convenient for many laboratories throughout the world (95–97). When testing a small number of unique designs, scientists typically order the DNA sequence that encodes the protein-of-interest from a commercial vendor, which must be cloned into an expression vector that harbors signal peptides, affinity tags, among other features. Ordering full-length genes of glycan-masked constructs may be prohibitively expensive when ordering dozens or even hundreds of variants (95). However, depending on the complexity between constructs, it may be substantially more efficient in terms of time to order full-length genes of, say, hyperglycosylated constructs. Additionally, other mutations that accompany the glycoprotein may be necessary. Thornlow and colleagues (20) cloned five distinct genes when designing hyperglycosylated hemagglutinin constructs with up to ten engineered glycosylation sites, totaling 16 *N*-glycosylations, plus four cysteine mutations that occluded undesired trimer interface epitopes by ‘locking’ the trimeric structure through disulfide bonds between monomers. Similarly, unique strains of viruses may differ drastically from one another, but common epitopes may be desirable for immunofocusing. Brinkkemper and colleagues (98) demonstrated an alternative to chemical synthesis of template DNA. They isolated five strains of HIV, each widely deviating in phylogeny, directly from patient samples. Brinkkemper et al. then supplemented Env proteins’ native glycan shields with engineered glycosylation sites to block immunodominant but nonprotective epitopes. In the study, several mutations were further introduced to stabilize the engineered Env variants, and were also integrated into a two-component nanoparticle system.

An alternative to purchasing full-length genes of glycosylated proteins and immunogens is site-directed mutagenesis (SDM) in which the wild-type gene is mutated to present sequons for glycosylation (**Figure 3**). Polymerase chain reaction (PCR) allows specific amplification or modification to template DNA *in vitro*, and it has made SDM convenient and affordable (25, 99). Short (20 to 60 nucleotides), single-stranded DNA primers can be used to amplify site-specific mutants through PCR (100). Xu et al. (101) screened 16 glycosylation mutants and found that seven expressed. From this initial screen, Xu and colleagues (101) combined the four most desirable glycan mutants into constructs with up to four glycosylation sites that expressed well, without the need to order new, full-length genes. Alternatively, larger glycoprotein mutant libraries can be generated and screened at once through cell-surface display (102). These methods, however, may be more suited for *in vitro* stabilization of glycoproteins through directed evolution rather than engineering of novel glycosylation sites (103). This is because of the relatively low frequency of mutation during error-prone mutagenesis that is highly unlikely to yield the specific NxT/S motif if it was absent in the native sequence. Regardless, SDM makes introduction of the glycosylation sequon into wild-type sequences relatively trivial at the genetic level, but does not guarantee successful glycosylation when expressed. It is worth noting the potential utility of *in vitro* engineering techniques, as further stabilization or functional modification of glycan-masked immunogens may be necessary for some projects (104).

### 4.2 High eukaryote expression systems

High eukaryote-based expression systems, particularly those of mammalian cell origins, prevail in the literature for production of designed glycoprotein immunogens, especially for viral immunogens. Mammalian cells efficiently *N*-glycosylate and fold complex proteins that simpler expression systems, such as bacteria, are not equipped to handle (91, 105). Additionally, the environment of mammalian cells is biologically relevant to the environment in which wild, pathogenic human viruses replicate. Thus, mammalian expression cell lines enable recombinantly expressed viral proteins to adopt native-like trimerization, cysteine pairing, host-like glycosylation, and are subject to the protein folding quality-control mechanisms of the endoplasmic reticulum, and secretion into the extracellular matrix. The recombinant expression of glycoproteins *in vivo* is essentially identical to expression of non-glycosylated proteins (106). The major distinction between non-glycosylated and *N*-glycosylated protein production in eukaryotes is the presence and class of the signal peptide encoded in the plasmid that facilitates trafficking of the translated protein into the endoplasmic reticulum, where the initial glycan is ligated, and then to the golgi apparatus for glycan maturation (91, 98). Care must be taken when selecting the secretion tag, as trafficking is dependent on the organism used for protein expression (93). Additionally, each propeptide will have unique trafficking properties regarding where the fused protein is localized, and what other, if any, post-translational modifications are performed (107–109).

Several human cell lines have been optimized for recombinant protein expression. The most ubiquitous of these, in the context of glycan masked immunogens, are human embryonic kidney (HEK) cells of the 293 lineage (91, 106). HEK293 clonal lineages, such as Gibco Expi293F™, have been engineered for faster growth rates, greater recombinant protein yields, and can be non-adherent to facilitate increased growth densities and scales (52, 106). Though these features are attractive on-paper, in practice they may adversely affect glycoprotein homogeneity and stability. High growth densities and prolonged expression times may influence glycan identity and protein stability due to nutrient and/or oxidative stress, and other unforeseen biological phenomena (110). It is thus prudent for researchers to optimize and investigate the effects of expression time on their proteins’ quality. Non-human mammalian cells, such as Chinese Hamster Ovary cells, are more commonly used for high-yield production of proteins due to their high suspension growth densities, and being less susceptible to contamination from human pathogens (106). CHO cells do vary in glycosylation patterns, including several non-human glycan residues and even some glycans with immunogenic potential, such as α-galactose (111). Despite this, nearly 70% of available biopharmaceuticals are produced in CHO cells, mostly due to favorable large-scale production properties such as high productivity, processivity, and being less susceptible to contamination (105). However, with regards to vaccine production, cells of human origins are generally favored due to the biological relevance of the immunogens produced. It is important to note that bacterial immunogens may not be suitable for production in mammalian cells, as these tend to be toxic. In addition, most pathogenic bacterial proteins are not natively *N*-glycosylated, and mutation of native, incidental NxT/S sequons that were not intended for glycosylation may be required. Regardless, the versatility of mammalian cell expression systems has enabled scientists to establish both high-throughput expression and screening workflows (112), and high-yielding protocols for recombinant glycoprotein production and characterization (113). Insect (25, 114) and plant cells (115–117) have also been reported in the use of producing glycoprotein immunogens, though these are sparse in comparison to mammalian cell lines regarding pharmaceuticals, mostly due to the presence of harmfully immunogenic glycan residues in these organisms. While they currently remain relatively unpopular in the realm of vaccine production, we speculate that successful humanization of glycosylation patterns within some plants, namely *Nicotiana benthamiana*, and/or other organisms may facilitate large-scale expression workflows more conducive to biopharmaceutical production.

### 4.3 Alternative glycoprotein expression systems

Mammalian cell facilities can be prohibitively expensive and, in most cases, demand specialized equipment, training, and often demand increased biosafety clearance for laboratories to operate (118). Thus, some laboratories may require more accessible organisms to cultivate such as fungi or prokaryotes (119). Additionally, transfection into mammalian cells requires that the plasmid DNA be of high quality and quantity, and may require specialized reagents (120–122). The preparation of sufficient DNA for transfection, although well established, can be a bottleneck in throughput for experiments (123). Simpler organisms such as yeast and prokaryotes offer less rigorous methods of transformation, such as heat-shock, that require only basic laboratory chemicals and equipment (124–126). Additionally, these organisms offer faster growth rates and cheaper reagents to facilitate growth compared to high-eukaryotes. Thus, although organisms such as yeast and bacteria deviate from humans regarding glycosylation patterns, or even glycocompetence, their convenience and accessibility alone may offset their disadvantages in some experiments, particularly in the prototyping phase. We must emphasize that yeast and prokaryotes are best utilized in the design phases, since their glycans vary drastically in composition, branching, occupancy, and especially immunogenicity (127). Thus, mammalian cells are still recommended for the final stages of glycoimmunogen characterization. Such an approach has been used to screen immunogenic libraries through yeast display followed by production of hits in mammalian cells (128). This allows for a time and cost-efficient pipeline that still allows for experimentation on vaccine candidates produced in biologically relevant environments. Ultimately, it is at the discretion of the scientist to weigh such advantages and disadvantages when designing their experiments (see **Figure 3**).

Among the many species of yeast, two stand out as the most common in laboratories: *Saccharomyces cerevisiae*, and *Pichia pastoris* (129). Laboratory yeast strains can grow densely with affordable reagents and reliably with minimal equipment. However, yeast glycosylation is known to be mannose-favored, which deviates greatly from human glycosylation patterns (130, 131). In addition, induction of yeast in protein production may require methanol, which is expensive, toxic, and may require additional chemical safety clearance (129). Thus, while yeast can be a useful production medium for both the prototyping phase and for large-scale production, yeast’s hypermannosylation is known to alter immunogenicity, which can confound the design of glycan-masked vaccine candidates. *P. pastoris* has been used to produce a glycosylated immunogen based on the stem domain of Influenza hemagglutinin (132). In this work, Wang and coworkers (132) demonstrated the use of *P. pastoris* expression through both shake-flask culturing and bioreactor cultivation. They reported protein yields of 10 mg/L through shake-flask and 100 mg/L in fed-batch cultivation. Additionally, Burnap and colleagues (133) used *P. pastoris* to produce the Abdala vaccine for SARS-CoV-2, which itself is based on the receptor-binding domain (RBD) of the spike protein. The scalability of yeast systems are showcased here, as Burnap and coworkers (133) acquired their samples from 75L and 3,000L lots of the Abdala vaccine. We note that the SARS-CoV-2 Spike RBD construct used by Burnap and colleagues is relatively small at about 26 kDa without glycans, and highly stable. Therefore, other constructs may vary in success with respect to production scalability. *P. pastoris* has shown use for immunogen production in other SARS-CoV-2 vaccine constructs (134–136). *S. cerevisiae* tends to see more use in immunogen engineering through yeast display (137, 138), but is also popular for production of other biopharmaceuticals (139). An extensive review showcasing the versatility of yeast in production of vaccines and pathogen-associated proteins up to 2018 has been reported elsewhere (140).

Bacterial organisms, namely *Escherichia coli*, offer some of the fastest growth rates among model laboratory organisms, high growth densities, and are highly affordable and accessible (92). Additionally, they are relatively easily genetically engineered and many unique strains have been made commercially available for a wide variety of niche applications (141). However, most prokaryotes, including *E. coli*, lack the complex quality control of protein folding and *N*-glycosylation offered by Eukarya. Prokaryotic organisms may, thus, seem to be poor choices for production of glycoproteins at first glance. However, as Keys and Aebi (142) pointed out in their review on this subject, this is not an inherent weakness, and has presented an opportunity to engineer glycosylation pathways that can *N*-glycosylate exogenously expressed proteins in a bio-orthogonal manner. For this to occur, machinery from an *N-*glycosylating organism must be produced alongside the protein-of-interest (143, 144). One such example is the oligosaccharyltransferase PglB from the bacteria *Campylobacter jejuni.* While C. jejuni glycosylates with GalNAc-based heptasaccharides that are known to be immunogenic, work has been done on modifying the glycans into more eukaryote-like GlcNAc glycans (143). Furthermore, the glycoforms produced by C. jejuni are usually homogenous, which may be advantageous for, e.g. biophysical experiments but do not simulate the heterogeneity of native eukaryotic glycosylation. It must be noted that the *N-*glycosylation sequon in *C. jejuni* deviates from that of eukaryotes, as it is D/E-X-N-X-S/T, where X is any amino acid besides proline, with DQNAT being the optimal acceptor sequence for PglB glycosylation (145). The orthogonality of engineered *E. coli* glycosylation has been heavily exploited in the production of glycoconjugate vaccines against bacterial pathogens, wherein carrier proteins are *N-*linked to pathogen-associated bacterial glycans (146). These carrier proteins, however, are typically established and stable, and are not themselves the immunogenic target. The limiting factor in bacterial production of glycoimmunogens may instead be the complexity of the antigens of interest themselves, as opposed to the glycocompetency of *E. coli* (105). The primary challenge in producing redesigned glycoprotein immunogens in bacteria would most likely be solubility, as many viral fusion protein targets are metastable (147). Incompatibilities with the *C. jejuni* sequon may also conflict with a researcher’s designs.

Cell-free protein synthesis (CFPS) is a rapidly expanding technology in both popularity and utility. A review on vaccines produced through CFPS has been made available by Hu and Kamat (148), and others are available for glycoproteins (149, 150). Briefly, CFPS utilizes the biosynthetic machinery of an organism of choice and is supplemented with a mixture of biochemical reagents (151). A genetic template is then introduced to the mixture, wherein the desired protein is produced *in vitro*. The speed, modularity, and throughput of CFPS systems are what make CFPS an attractive option when screening designed protein sequences. CFPS components are relatively affordable and easy to acquire, and reactions as small as 10 µL can yield sufficient protein for simple assays such as enzyme-linked immunsorbance assays (ELISA) (152, 153). Adiga and colleagues reported a portable system for the rapid production and purification of therapeutic proteins using a CHO-based lysate system which was supplemented by purified microsomes to facilitate glycosylation (154). Through this, they produced the glycosylated therapeutic protein erythropoietin, the vaccine immunogen diphtheria toxoid DT5, and other therapeutic proteins. It must be noted that eukaryote-based CFPS systems require supplementation with microsomes to glycosylate efficiently (155). Counterintuitively, CFPS with prokaryotic lysates for glycoproteins may offer the highest degree of control in terms of glycan identity and homogeneity. DeWinter and colleagues recently reported the development of cell-free glycoprotein synthesis (CFGpS) through mutant PglB-expressing *E. coli*-derived extracts that are capable of exclusively linking recombinant proteins to GlcNAc (156). This study demonstrates promise in terms of homogeneity of glycoforms for target glycoproteins, but suffers from similar shortcomings to that of *in vivo* bacterial expression. Given the novelty of this study, it is unclear how robust this system, or CFPS in general, will be for immunogens and overall vaccine production, though other, similar platforms have been used for various therapeutic glycoproteins (112, 140, 144, 149). The inherent stability of the protein-of-interest is likely to be the limiting factor in terms of expressibility in CFPS.

## 5 Experimental techniques: glycan verification

Recombinant glycoprotein purification is a vast field in itself and has been extensively covered elsewhere (157). The next step from acquiring pure glycoprotein samples is to evaluate whether or not the engineered sequon is properly glycosylated. The presence of the sequon for *N*-glycosylation (NxS/T) does not inherently guarantee glycosylation. Thus, it is essential to experimentally verify the presence of glycans, which can be done through various techniques: SDS-PAGE, immunoblotting, enzymatic cleavage of the sugar and mass spectrometry. A summary of the publications using these techniques is shown in **Table 3**.

**Table 3:** List of publications using experimental techniques to verify the presence of glycans on the immunogens.

Technique	Publication	Details
SDS-PAGE/Western	Garrity RR. et al., 1997 (33)	Both
	Pantophlet R. et al., 2003 (12)	Both
	Selvarajah S. et al., 2008 (22)	Both
	Lin SC. et al., 2012 (24)	Both
	Lin SC. et al., 2014 (25)	Both
	Chen TH. et al., 2019 (34)	Both
	Sampath S. et al., 2013 (29)	Both
	Forsell MNE. et al., 2013 (23)	SDS-PAGE only
	Eggink D. et al., 2014 (28)	Both
	Ingale J. et al., 2014 (16)	SDS-PAGE only
	Du L. et al., 2016 (185)	SDS-PAGE only
	Tai W. et al., 2019 (31)	Both
	Boyoglu-Barnum S. et al., 2020 (19)	SDS-PAGE only
	Chen TS. et al., 2021 (26)	Both
	Adolf-Bryfogle J. et al., 2024 (32)	SDS-PAGE only
PNGaseF/EndoH	Garrity RR. et al., 1997 (33)	PNGaseF only
	Lin SC. et al., 2012 (24)	Both
	Lin SC. et al., 2014 (25)	PNGaseF only
	Chen TH. et al., 2019 (34)	Both
	Sampath S. et al., 2013 (29)	PNGaseF only
	Forsell MNE. et al., 2013 (23)	PNGaseF only
	Ingale J. et al., 2014 (16)	PNGaseF only
	Boyoglu-Barnum S. et al., 2020 (19)	PNGaseF only
	Chen TS. et al., 2021 (26)	PNGaseF only
	Adolf-Bryfogle J. et al., 2024 (32)	PNGaseF only
Mass Spectrometry	Ingale J. et al., 2014 (16)	MALDI-TOF
	Du L. et al., 2016 (185)	MALDI-TOF
	Duan H. et al., 2018 (17)	LC-MS

The first column reports the technique name, the second column reports the publication references and the third column reports additional details.

### 5.1 SDS-PAGE and western blotting

Sodium Dodecyl Sulfate Polyacrylamide Gel Electrophoresis (SDS-PAGE) is commonly used to differentiate proteins based on their size (158). Denaturing agents (such as SDS) and reducing agents (such as DTT or β-mercaptoethanol) lead to complete unfolding of the proteins in the mixture, which are then loaded into a polyacrylamide matrix. The denatured proteins migrate at different speeds based on their molecular weight. However, it should be noted that glycosylation often produces diffuse, smeared bands due to glycan heterogeneity, which can complicate interpretation. In combination with SDS-PAGE, Western blotting is used to validate the presence of a protein of interest—for example, via detection of a polyhistidine tag (159). Antibodies specific to the tag and/or to the protein of interest are used to target and identify the corresponding bands within the mixture. Nevertheless, it should be noted that epitope tags may be masked or altered by glycosylation, potentially reducing antibody binding. These techniques are also widely used to identify glycosylated variants in natural glycoprotein and in glycan masking. Since glycosylation is a covalent modification, denaturing and reducing agents do not remove the glycans from the protein, but the increase in weight is detectable via SDS-PAGE. Since protein glycosylation in the endoplasmic reticulum and Golgi is a stochastic process, glycan heterogeneity is obtained and the additional molecular weight ranges from 0.5 kDa to 2.5 kDa (160). This shift is typically visible on 10%, 12%, or 15% acrylamide SDS-PAGE gels. Glycan-specific antibodies are available, but they are often very specific for sugar type, modification, and overall chemistry (161). Given that these antibodies are specific to glycans and not the POI, non-specific detection of glycans should be expected in unpurified protein samples.

Testing glycosylated immunogens by SDS-PAGE is a simple and cost-effective method to indirectly demonstrate glycan presence. It is often used in glycan masking experiments either as a standalone technique or in combination with Western blotting (see **Table 3**). However, this method is low resolution, and when multiple glycans are present on the same protein, additional techniques may be required to identify glycoforms in samples. SDS-PAGE cannot distinguish between glycan occupancy vs. glycan composition, nor can it resolve glycoforms differing by <1 kDa. Alternative techniques may be required to identify glycoforms in samples.

### 5.2 Enzymatic cleavage

Enzymatic cleavage of glycans is a very common technique to verify that glycans are present on the immunogen when performing glycan masking. In particular, the protein of interest is treated with specific glycosidases, enzymes which cleave glycans at points along the glycan chain (162). Following the deglycosylation of the protein, SDS-PAGE and Western blotting enable verification of the deglycosylation by comparing molecular weights and protein-specific identification via epitope tags (163, 164). These experimental methods enable rapid and inexpensive qualitative verification of protein glycosylation, though are not robust when identification of glycans and homogeneity is desired.

**Figure 4:**
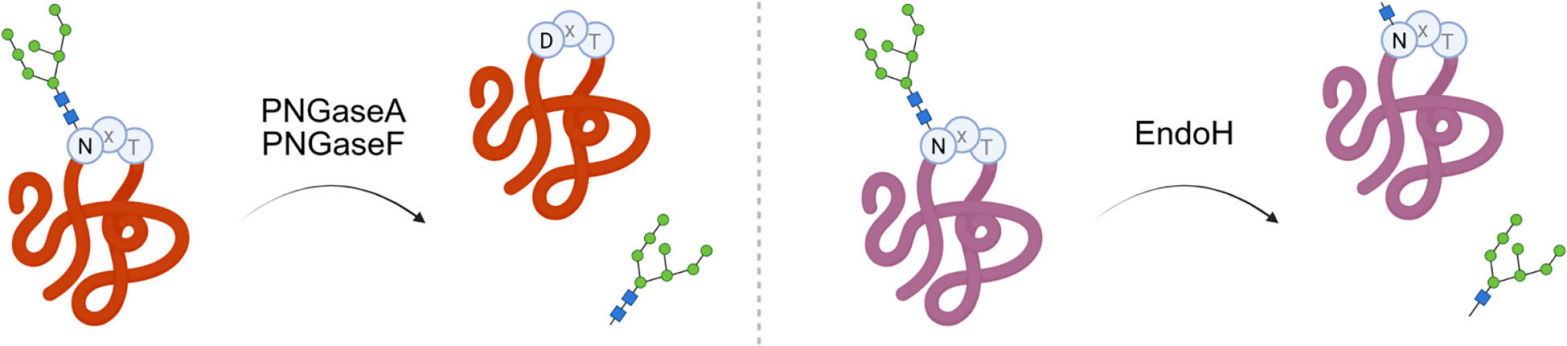
Glycosydases for glycan removal. Representation of glycan cleavage catalyzed by peptide:*N*-glycosidases (left) and endo-glycosydases (right). PNGases will remove the full sugar and change the asparagine (N) to aspartic acid (D), while EndoH will leave one sugar attached to the protein asparagine (N). Created in BioRender. Martina, C. (2026) https://BioRender.com/ugyhecw.

Different glycosidases are key for the cleavage of the glycosidic bond to separate glycans from their proteins. The most common enzyme used to validate glycan presence on the protein in glycan masking for immunogen design is Peptide:*N*-glycosidase F (PNGase-F) (165). This enzyme is very versatile, cleaving the glycosidic bonds between the innermost N-acetylglucosamine and asparagine residue of N-linked glycans as shown in **Figure 4**. PNGaseF has the advantage of leaving the separated oligosaccharide intact, which can then be analyzed and identified through mass spectrometry. To note, after cleavage of the sugar, PNGaseF will change the asparagine (N) to aspartic acid (D), as shown in **Figure 4**. This is very important to take into account if performing antibody binding experiments with the sequon but without the sugar itself. Binding to the antibody might be affected by the change from N to D due to PNGaseF cleavage.

Though PNGaseF is widely used, it is unable to cleave N-linked glycans with α-1,3 core fucosylation, which is commonly found in N-linked glycans of plants (166). PNGaseA exhibits the same enzymatic activity as PNGaseF, but it is not inhibited by the presence of an α 1,3 core fucosylation and it can cleave more complex glycans. Endoglycosidase H (Endo H) is an additional glycosidase with higher specificity compared to PNGaseF, as it only removes high-mannose or certain hybrid N-linked glycosylations (167). It cleaves the beta-linkage in between two N-acetylglucosamine residues, leaving one sugar attached to the protein (see **Figure 4**).

Small molecule glycosidase inhibitors such as Kifunensine and Swainsonine can be used during transfection to increase sensitivity to Endo H without compromising protein yield (168). The use of chemical inhibitors in glycoprotein studies is suggested as a cost effective and experimentally simple way to address certain issues that are inherent to studying glycoproteins such as the need to effectively remove glycans for crystallization or create greater homogeneity of glycoforms. Glycosidase inhibitors have also been used to optimize monoclonal antibody production and function. Li et al. discusses cases where these inhibitors have been used to glycoengineer mAbs as a way to enhance their therapeutic activity. They also suggest their potential use in addressing species dependent variations in N-glycan processing of mAbs (160). Kifunensine in particular has also been used to study the role of glycans in regulating viral entry (169). However, it should be noted that Kifunensine can induce high-mannose glycosylation and may artificially increase epitope shielding, potentially altering immunogenicity readouts. On the other hand, glycosidase inhibitors can be used strategically to control the glycan forms presented by the immunogens, for example through mannose enrichment. Finally, cheaper chemical methods to remove glycans from proteins (i.e. sodium hypochlorite, NaOCl) exist, though they are less efficient than enzymes (170), and the harsh conditions might damage and unfold the protein. Glycosidases are experimentally useful, but they are often paired with other experimental methods which help verify the successful removal of the glycans, maintenance of the protein’s structure, and identification of the removed glycans.

### 5.3 Mass spectrometry

Mass spectrometry (MS) is a powerful analytical tool for evaluating glycosylation sites and determining the structures of the glycans (171). The analysis of N-glycopeptides through MS presents several obstacles. First, the structural heterogeneity of glycans, in terms of composition, branching, and linkage, complicates MS analysis, as each glycosylation site on a peptide can be occupied by various oligosaccharide structures. Additionally, the increased acidity and negative charge of glycosylated peptides reduce their ionization efficiency, particularly when their MS signals compete with a much larger number of non-glycosylated peptides that produce stronger ion currents (172). Consequently, their MS signals appear considerably weaker than those from non-glycosylated peptides. Enrichment of *N*-glycopeptides before MS analysis becomes important to enhance their detection sensitivity (173). Lectin-based affinity enrichment utilizes the selective binding between lectins and sugar moieties present on glycopeptides (174). Hydrophilic interaction chromatography relies on polar interactions occurring between glycan hydroxy groups and the stationary phase; this method has gained widespread adoption for its effectiveness in uniformly enriching *N*-glycopeptides (175). Chemical covalent interaction relies on forming covalent bonds between specific functional groups on the glycan structures and chemically modified solid supports or beads (176). Given the structural diversity among glycoforms and the heterogeneous nature of *N*-glycosylation sites, there is no single universal *N*-glycopeptide enrichment strategy, and depending on each study, the appropriate method of choice is selected with further optimization (177).

Regarding MS analysis following glycopeptide sample preparation, matrix-assisted laser desorption/ionization mass spectrometry (MALDI-MS) is the most commonly used technique. The workflow involves enriching glycopeptides, treating them with PNGase-F for deglycosylation, then analyzing the resulting free peptides. This enables identification of *N*-glycosylation site locations and the individual peptides. The masses of released glycans can also be used to deduce their structural information (178–180).

Glycopeptides can also be characterized using electrospray ionization (ESI)-based LC-MS/MS strategies. In this approach, glycopeptides are first separated by liquid chromatography (LC) based on their hydrophobicity and glycan composition, then ionized under gentle electrospray conditions to preserve labile glycan structures. The resulting ions are analyzed in the mass spectrometer, where tandem MS (MS/MS) fragmentation provides information about both the peptide backbone and attached glycans. This method enables identification of glycosylation sites and more precise glycan structural information, including stereochemistry of glycosidic linkages and branching patterns (181, 182).

Overall, with the improvement and evolution of MS technology and sample preparation techniques, MS has made great strides in glycoprotein analysis. Contemporary applications now encompass glycopeptide sequence determination, glycosylation site occupancy, and comprehensive glycan identification.

## 6 Experimental techniques: functional, and conformational studies

Protein glycosylation and hyper-glycosylation may lead to either global or local misfolding. In the former case, the protein’s structural integrity is compromised by the presence of the glycan(s) and protein misfolding occurs in the ER and/or Golgi (183). In these situations, the protein itself is unlikely to mature and thus be extracted in high quantities. In the case of local misfolding, glycosylation may partially alter folds proximal in space to the glycosylation site. This may pose a risk in the development of vaccine candidates, since altered conformations could elicit antibodies able to recognize the designed immunogen but not the native viral protein itself (184). One avenue to verify that engineered glycans do not affect target immunogen folding is by assessing functional activity. In some cases, however, immunogenic properties may be more important than maintaining the immunogen’s native function and may even demand inactivation of the function while maintaining the overall structure. Determination of the molecular structure of engineered glycan-masked immunogens reveals the conformational integrity of the antigen, usually unambiguously (6). An overview of examples for verification of structural and functional integrity of glycan masked immunogens is presented in **Table 4**.

**Table 4:** List of publications for structural and functional studies of glycosylated immunogens.

Technique	Publication	Method
Structural validation	Garrity RR et al., 1997 (33)	Binding to antibodies and receptors
	Pantophlet R. et al., 2003 (12)	Binding to antibodies
	Pantophlet R. et al., 2004 (13)	Binding to antibodies
	Selvarajah S. et al., 2005 (14)	Binding to antibodies
	Ahmed FK. et al., 2012 (15)	Binding to antibodies
	Selvarajah S. et al., 2008 (22)	Binding to antibodies, electron microscopy
	Sampath S. et al., 2013 (29)	Binding to receptors
	Forsell MNE. et al., 2013 (23)	Binding to antibodies and receptors
	Eggink D. et al, 2014 (28)	Binding to antibodies
	Ingale J. et al., 2014 (16)	Binding to antibodies and receptors
	Du L. et al., 2016 (185)	Binding to antibodies and receptors
	Duan H. et al., 2018 (17)	Binding to antibodies, electron microscopy
	Bajic G. et al., 2019 (18)	Luminex assay
	Thornlow DN. et al., 2021 (20)	Binding to antibodies
	Tai W. et al., 2019 (31)	Binding to antibodies
	Boyoglu-Barnum S. et al., 2020 (19)	Binding to antibodies, electron microscopy
	Chen TS. et al., 2021 (26)	Binding to antibodies
	Adolf-Bryfogle J. et al., 2024 (32)	Electron microscopy
	Lin WS. et al., 2021 (99)	Binding to antibodies
Functional activity	Garrity RR et al., 1997 (33)	Viral replication
	Wanzeck K. et al., 2011 (206)	Viral replication
	Lin SC. et al., 2012 (24)	Hemagglutination activity
	Sampath S. et al., 2013 (29)	Cytoadherence assay
	Lin HH. et al., 2019 (21)	Flagellin activity
	Chen TS. et al., 2021 (26)	Hemagglutination activity

The first column reports the technique name, the second column reports the publication references and the third column reports additional details.

### 6.1 Structural validation

In most cases, it can be assumed that a glycan-masked protein has folded correctly if target antibodies bind (**Table 4**). This is only the case for conformational epitopes, where epitope residues are proximal in space but not in sequence, thus binding depends on the immunogen’s folding. However, certain caveats must be considered: 1. Glycan masking may sterically prevent antibody access even if correctly folded. 2. Some antibodies tolerate partial misfolding. For some projects, known antibodies against desirable conformational epitopes are not available, thus proper folding cannot be confidently deduced from antibody binding, necessitating molecular-level structural determination. However, these methods are generally non-trivial, as they can be highly time-intensive, demand specialized equipment and training, and typically require protein samples that are of superb quality. Still, performing structural validation may be critical to evaluating novel, engineered glycan-masked immunogens.

Low-resolution (with respect to structure) but high-throughput validation of structural integrity is commonplace among designed glycan masked immunogens. ELISAs are biochemical, colorimetric assays that use antibodies for capture and detection of target proteins. They are among the most common antibody-antigen detection methods due to their affordability, convenience, and versatility, and do not require advanced laboratory equipment. ELISA has been used extensively for preliminary validation of designed glycan-masked immunogens such as HIV Env (12–15, 17, 22, 23, 33, 64), influenza HA (19, 20, 26, 28), Zika virus envelope protein (31), Ebola virus GP (101), and MERS/SARS Spike protein (73, 185). Biolayer interferometry (BLI) is a biophysical assay that grants real-time kinetic data on binding, can detect affinity reductions due to glycan occlusion, even when structure remains intact, and has also been used for a variety of immunogenic targets (16, 64, 186–188). Both methods can be label-free and can also support qualitative collection of binding data with crude protein in clarified cell lysates or media, therefore supporting high-throughput workflows (189, 190), yet they cannot verify epitope geometry. Flow cytometry has also been used and is popular for cell-based assays (17, 18, 29, 185, 186), though this route may demand covalent fluorescent labeling to the protein-of-interest which may adversely affect protein integrity (191). This obstacle may be overcome via site-specific labeling methods such as sortase tagging (192). Still, flow-cytometry provides an extensive range of data when characterizing cellular responses to immunogens *in vitro* (193–196). Other methods such as luminex assays (18), differential scanning fluorimetry (64, 101, 187), AlphaScreen (185, 196), surface plasmon resonance (17), and circular dichroism spectroscopy (36), among others, have been used towards the same end but are not as frequently reported.

Molecular structure determination of glycoproteins is a paradoxical issue with regard to known techniques. The elucidation of protein structures is most popularly done through cryogenic electron microscopy (Cryo-EM), x-ray crystallography (XRC), or nuclear magnetic resonance (NMR). Put simply, the resolution obtained from the former two rely on the rigidity of the analyte, as they are measurements of electron densities (197). Resolution is lost in more flexible regions, such as glycans and flexible loops, where electron densities may not conform. NMR is the opposite, as its signal relies on the resonance, i.e. free movement, of nuclei, but sample preparation demands isotope labeling (198). In our review, we have found that, of the studies that experimentally validate the glycan-masked immunogens’ molecular structures, Cryo-EM is the most popular route. This is likely due to the robustness of Cryo-EM in analyzing heterogenous samples, such as those of glycoproteins, in addition to quick data collection compared to other methods like XRC (197, 199). Cryo-EM tends to perform best on large proteins and complexes, ideally exceeding 100 kDa, but has been shown for complexes down to 50 kDa (200, 201). Berndsen and colleagues (5) demonstrated the use of Cryo-EM in evaluating the molecular topology of glycan-masked HIV-1 Env, lending insight into potentially accessible pockets of the Env protein. In addition, Cryo-EM can be amenable to relatively high-throughput, low-resolution structural evaluation of constructs through negative-stain cryo-EM (98, 101, 199, 202). Cryo-EM is thus particularly useful when structurally characterizing protein nanoparticle constructs, including designed hyperglycosylated constructs (17, 19, 32, 58, 59). XRC has shown use in glycan-masked immunogen design (18) and for many viral glycoproteins, though heterogenous samples may interfere with crystallization (203, 204). NMR may be more suited for the characterization of glycan residues (205).

### 6.2 Functional activity

The simplest way to verify whether engineered glycosylation affects viral integrity is to assess viral replication with the mutants. This has been done by Garrity and team with HIV (33), who generated nine viral variants inserting from one to four glycosylation in the full length genome of HIV-1. They then compared the replication and infectivity rates of each variant, discovering that only one mono-glycosylated variant had rates comparable to the ones of the WT virus. Three other monoglycosylated variants and one biglycosylated variant show a slower growth but similar infectivity, and the remaining bi- tri- and tetra- glycosylated variants were highly affected by the glycan presence for both growth and infectivity. Similarly, Wanzeck and coworkers (206) tested their two variants of influenza virus (bearing two and four additional glycans) for replication efficiency, confirming that viral replication wasn’t affected by glycosylation.

Another method to test functional activity and protein integrity after glycosylation is to use known functions of the antigens of interest. For example, influenza HA binds sialic acid exposed on blood cells, which clump together, resulting in hemagglutination (207). Thus, a hemagglutination assay can be used to verify the structural and functional integrity of glycan masked versions of HA. This has been done by Lin and team who tested eight single glycan mutants of HA H5N1 for hemagglutination activity, of which six variants retained functional activity, and two failed to agglutinate red blood cells (24). Similarly, Chen and team, tested for hemagglutination activity three adenovirus vectors encoding for HA glycosylated variants, showing that the mutants were functionally similar to the wild-type (26).

Similarly to the HA of influenza virus, the malaria *P. vivax* antigen Duffy binding protein (PvDBP) binds to the Duffy receptor also exposed on the surface of red blood cells. Its functional activity can be tested *in vitro* with an established cytoadherence assay: the antigen is considered fully functional when five or more red blood cells bind to the cells overexpressing PvDBP and GFP (208). Sampath and coworkers tested three of their PvDBP glycan mutants presenting one, three and eight additional glycans and saw that, compared to the wild-type protein, binding was reduced to 4% in one candidate and to 75% and 88% in the remaining two (29).

Functional assays for glycan masked immunogens are important, since single glycosylations can disrupt the antigen activity and/or viral replicability. However, this approach is not always possible, since many pathogenic antigens might lack specific activities that could be used in parallel to structural validation. Additionally, unlike many structural approaches, functional assays are not generalizable and depend on the pathogen in question.

## 7 Discussion

Glycan masking is a powerful method in epitope-focused vaccine design and is gauging considerable interest in the field of reverse vaccinology. Among others, its main applications in the field of immunogen design are immuno-focusing, immuno-shifting, immuno-broadening, and immuno-altering (8). Here, we focus on computational and experimental methods to design immunogens through the addition of N-linked glycosylations (42).

Many computational methods are available to predict glycosylation sites as well as insert sequons for subsequent glycoprotein modeling. Tools that analyze sequences for potential glycosylation sites based on homologous sequences are available (18, 24) in addition to glycosylation predictors that identify sequons from the protein sequence (48) and structure (49–51). More advanced tools utilize machine learning and allow for the prediction of other post-translational modifications (209). In addition to sequon predictors and tools used for sequon insertion, recent advances in both structural biology and AI have allowed for the creation of tools for glycoprotein modeling, which can typically insert and subsequently model glycosylation computationally. These tools can be broadly separated into physics-based and AI-based. Physics-based glycan modeling examples include the GlycanTreeModeler pipeline within the RosettaSuite and Glycosylator (32, 52, 55). Additionally, computational tools based on MD pay great attention to the development of forcefields that accurately predict glycosylation and glycan pose over the course of a simulation (68–71). Lastly, AI approaches have played a large role in recent years in the field of glycoprotein modeling as it relates to immunogen design. Tools such as AlphaFold (53, 76), ColabFold (77), RFAA (54), and others utilize AI to allow for accurate, easy-to-use, and rapid glycoprotein prediction utilizing sequences as input. The trajectory of this field appears to be following the general trend of machine learning being incorporated into workflows. However, one of the major gaps in training glycan-based AI models is the relatively small and sparse datasets of glycoproteins and glycan conformations. Additionally glycans are flexible and often poorly resolved experimentally, affecting the quality of the available datasets. Sequon and glycosylation predictors can help in the computational design, but none can predict whether the engineered site will actually be glycosylated in a given expression host and whether the glycosylation will disrupt folding, trafficking, or immunogen stability. Physics-based methods remain valuable with regard to modeling glycoprotein structures and interactions, and with a more extensive collection of *in silico* and *in vitro* glycan masking data, new AI model could be specifically trained on and for glycoprotein and glycan dynamics.

Once the glycoprotein structure has been predicted and assessed computationally, there are a number of techniques that can be used experimentally to synthesize these glycoproteins. For viral targets especially, mammalian cells are the most favored due to the biological relevance of the produced glycoproteins (91). Yeast make for well-rounded expression hosts as they are cost-effective, glycosylate natively, and are robust in small and large scale growths. The primary downside to yeast as expression hosts in glycan masking is the hypermannosylation of proteins. The large size of yeast mannose glycans may lead to false negatives when assessing target antibody binding. Bacterial immunogens may be best produced in glycocompetent/glycoengineered prokaryotes, as these are, in many cases, toxic to mammalian cells. Human-derived cell lines, such as HEK293, are the most representative expression hosts of how engineered glycans will affect target antibody binding. Beyond this, it is difficult to speculate which non-human expression hosts are best in mimicking the effects of human glycosylation patterns on target antibody binding. Each antibody/antigen complex will be unique and the natural heterogeneity of glycoforms already convolutes rational inference of the effects of glycan masking. This is also difficult to predict computationally, as running all possible iterations of designed glycan-masked immunogens in complex with every known glycoform of all common laboratory organisms is not feasible.

CFPS systems are also rapidly growing in popularity with recent use in vaccine production as reviewed by Hu (148). CFPS has several limitations, however, with one being the limited scalability of reactions. Increasing the CFPS reaction scale is complicated due to the need for pure biochemical components. CFPS systems of prokaryotic origins show some promise in the production of homogenous, human-like glycoforms, but have not yet been tested in the realm of immunogen design. These systems are still relatively early in their development, and we anticipate that the improvement and diversification of these systems may impact future production workflows based on cost and time effectiveness.

The explosion in available technologies have also enabled high-throughput approaches and applications for nearly all expression platforms (112, 129, 149), shifting the throughput bottleneck towards gene synthesis. While individual genes are affordable, screening a high number of genes can be costly. Several high-throughput commercial gene synthesis services (such as IDT eBlock or Twist BioScience) offer custom DNA synthesis at roughly $0.06-$0.07 per base. These costs rapidly add up, especially since most immunogens are far larger than 100 amino acids. The accessibility of artificial gene synthesis has seen drastic improvements throughout the decades but is now the limiting factor in high-throughput screening of proteins. Streamlining workflows in the chemical synthesis and purification of nucleoside reagents used in gene synthesis may improve downstream affordability.

After glycoprotein synthesis, experimental techniques are available for glycan verification with SDS-PAGE/Western Blot (160, 210, 211), PNGaseF/EndoH (165), and MS (171, 177, 179) being commonly used for this purpose. Blotting methods can be used to differentiate glycosylated and non-glycosylated variants based on their size, while enzyme based methods can be used to remove present glycans for evaluation. MS can be used in sequence determination as well as glycan identification and site localization.

Lastly, there are numerous techniques for functional and conformational study of glycoproteins. Structural validation can be done via lower resolution (with respect to structure) methods such as ELISA, BLI, and flow cytometry which are commonplace in the field of glycan-masked immunogen design. At residue-level resolution, Cryo-EM is perhaps the most robust choice when characterizing structures of engineered glycoproteins (201). However, the flexibility of glycans limits the usefulness of Cryo-EM in characterizing the potential mechanisms of antibody perturbation from engineered glycans. XRC can be used if atomic-level resolution is necessary, provided the glycoprotein samples are pure and glycoforms are homogenous. Additionally, NMR can be useful when used in the characterization of glycan residues (205). Comprehensive studies combining high-resolution structure data with binding assay datasets and computational simulations seem to be preferred when characterizing the effects of glycan masking. To our knowledge, there are no individual techniques or models that can accurately predict the extent to which glycans within and around epitopes-of-interest can obstruct antibody binding. AI-based methods may also be limited in use here, as datasets of glycoproteins are often limited in accuracy, size, and/or are incomplete. Even so, systematic placement of glycosylation sequons at rationally defined sites has led to extensive success in the realm of glycan masked immunogen design. This can be at least partially attributed to the relative simplicity of the NxT/S sequon, which enables scientists to infer the feasibility of glycosylation on solvent-accessible areas on the surfaces of antigens-of-interest. Precise identification of sites exposed on immunogen surfaces can be sufficient to narrow down the number of constructs tested. Subsequent filtering through other parameters, *e.g.* distance from the epitope, non-neutralizing epitopes, can also lessen the screening burden. Overall, glycan masking is a versatile and accessible strategy in immunofocusing, and readily compatible with nucleotide vaccine delivery platforms. We aim for this review to assist researchers from both computational and experimental backgrounds to enable the use of glycan masking in their research.
